# Online Schools and Children With Special Health and Educational Needs: Comparison With Performance in Traditional Schools

**DOI:** 10.2196/jmir.1947

**Published:** 2012-04-30

**Authors:** Lindsay A Thompson, Rick Ferdig, Erik Black

**Affiliations:** ^1^Department of PediatricsUniversity of FloridaGainesville, FLUnited States; ^2^Research Center for Educational TechnologyKent State UniversityKent, OHUnited States

**Keywords:** Virtual schooling, schools, K-12, children with special health care needs, online learning, education, adolescent health services, special education

## Abstract

**Background:**

In the United States, primary and secondary online schools are institutions that deliver online curricula for children enrolled in kindergarten through 12th grade (K-12). These institutions commonly provide opportunities for online instruction in conjunction with local schools for students who may need remediation, have advanced needs, encounter unqualified local instructors, or experience scheduling conflicts. Internet-based online schooling may potentially help children from populations known to have educational and health disadvantages, such as those from certain racial or ethnic backgrounds, those of low socioeconomic status, and children with special health care needs (CSHCN).

**Objective:**

To describe the basic and applied demographics of US online-school users and to compare student achievement in traditional versus online schooling environments.

**Methods:**

We performed a brief parental survey in three states examining basic demographics and educational history of the child and parents, the child’s health status as measured by the CSHCN Screener, and their experiences and educational achievement with online schools and class(es). Results were compared with state public-school demographics and statistical analyses controlled for state-specific independence.

**Results:**

We analyzed responses from 1971 parents with a response rate of 14.7% (1971/13,384). Parents of online-school participants were more likely to report having a bachelor’s degree or higher than were parents of students statewide in traditional schools, and more of their children were white and female. Most notably, the prevalence of CSHCN was high (476/1971, 24.6%) in online schooling. Children who were male, black, or had special health care needs reported significantly lower grades in both traditional and online schools. However, when we controlled for age, gender, race, and parental education, parents of CSHCN or black children reported significantly lower grades in online than in traditional schooling (adjusted odds ratio [aOR] 1.45, 95% confidence interval [CI] 1.29–1.62 for CSHCN, *P *< .001; aOR 2.73, 95% CI 2.11–3.53 for black children, *P *< .001.) In contrast, parents with a bachelor’s degree or higher reported significantly higher online-school grades than traditional-school grades for their children (aOR 1.45, 95% CI 1.15–1.82, *P *< .001).

**Conclusions:**

The demographics of children attending online schools do not mirror those of the state-specific school populations. CSHCN seem to opt into online schools at a higher rate. While parents report equivalent educational achievement in online and traditional classrooms, controlling for known achievement risks suggests that CSHCN and black children have lower performance in online than in traditional schools. Given the millions of students now in online schools, future studies must test whether direct assistance in online schools, such as taking individualized education plans into consideration, will narrow known disparities in educational success. Only then can online schools emerge as a true educational alternative for at-risk populations.

## Introduction

Asking about educational attainment in the primary care setting is common, since educational success is a culmination of children’s health and well-being. However, while much has been written, research and clinical interventions have not consistently narrowed educational disparities [1-6], mostly because there are few resources by which to quantitatively measure education as a health outcome. Educational outcomes for health are often relegated only to school days missed or appropriate grade level for age [7,8]. A relatively new educational innovation in the United States, kindergarten to 12th grade (K-12) online schooling, constitutes an online means by which children can maintain or further their educational progress. This Internet-based educational opportunity is ideally situated to providing an opportunity for improved educational and health outcomes and would allow for a centralized means to measure both health and educational progress.

The phenomenon of online schooling is not limited to the United States, although its definition and approach lack uniformity both internationally and across US states. For example, a variety of terms are used to describe online learning, including distance education, online schools, online learning, e-learning, and electronic learning. In general, however, the common understanding is that this type of learning simply takes place over the Internet [9]. Over a million US students participate, choosing online classes for a variety of reasons, including credit recovery, advanced preparation, schedule conflicts, home schooling supplementation, and the lack of local qualified instructors. Originating in the United States in 1995, state-funded online K-12 education now exists in 44 states [10,11]. Although school administrators, policy makers, parents, and students have questioned the effectiveness of K-12 online schooling compared with traditional, face-to-face schooling [12,13], numerous studies have documented evidence of their educational equivalence [14,15].

International efforts have developed similarly. In a recent survey of online education practices in 50 different countries, nearly 60% of respondents reported government funding for online programs at the primary and secondary school levels (5–18 years of age). Examples of growth and adoption include China’s online-schooling initiative, which has expanded from 1 institution in 1996 to more than 200 online schools, with enrollments exceeding 600,000 students. In British Columbia, Canada, approximately 12% of the student population participates in some form of online learning [9]. While more-developed nations (Australia, China, Denmark, Mexico, Canada, and the United Kingdom, for example) have more-advanced programs, online programs are emerging or have emerged in Africa (Egypt), Asia (Indonesia, Malaysia, Singapore, and Uzbekistan), Europe (Belgium, Finland, France, Germany, and Italy), Eastern Europe (Slovenia, Albania, Romania, and Serbia), the Middle East (Turkey and Israel), and South America (Argentina, Peru, and Uruguay) [9].

Both nationally and internationally, online schools have adopted many different models for course delivery to primary and secondary education students. Some offer the opportunity for students to earn a diploma and take all of their coursework online. Others only supplement traditional face-to-face schools. Course format also varies; some institutions allow students to self-pace, meaning the student is required to complete a requisite amount of work to earn credit for the course. Whether the student is able to do so in 6 weeks or 6 months is entirely up to the student. Other institutions may offer a format that is more traditional, in which the student has a fixed time during each school day to work through curricular content. It remains unknown, however, how online schooling may serve children from populations known to have health and educational challenges, such as those from certain racial or ethnic backgrounds, those with socioeconomic disadvantages, and children with special health care needs (CSHCN) [12,13,16,17]. Nonetheless, the potential advantages of online schools are substantial, with self-pacing and class attendance from home or even a hospital bed.

Given these potential advantages and the current popularity of online schooling, the purpose of this study was to describe and quantify who uses online schools and why. Drawing from parental survey results from three states, this study aimed to clarify four goals: (1) to establish a knowledge of the basic demographics of online-school users, (2) to gain an understanding of the educational background and success of online-school students, (3) to determine whether there is a high prevalence of CSHCN enrolled in online schooling, and (4) to determine how children perform in online schooling compared with their prior experiences in traditional school. Online schools may potentially allow US students known to have both educational and health challenges, such as those from certain racial or ethnic backgrounds, those with socioeconomic disadvantages, and CSHCN, to better succeed.

## Methods

### Survey Participants

We performed an observational study in three of the states that have established state-led online-school programs, all of which are in the southeastern region of the United States. A multidisciplinary team from the University of Florida, Colleges of Education and Medicine, contacted parents via email, with three sequential invitations, to participate in a brief, online survey that could be accessed via an embedded link. The three participant states and their state-led online schools were invited to participate from the 21 state participants in the Virtual School Clearinghouse [18], “a collaborative research project sponsored by the AT&T Foundation” that provides state-led online schools “with data analysis tools and metrics vital for school improvement.” Unlike cyber-charter schools or school district-oriented programs, state-led online schools are associated with state departments of education, which provides some similarity in the scope and nature of their operations.

**Figure 1:**
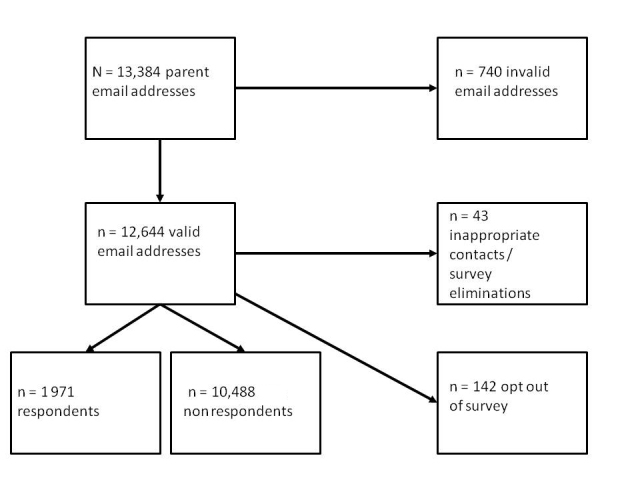
Progress of participants through the study.

 The three participating state-led online schools were required to supply email contact information for the parents of enrolled students. Using only these email addresses, this study achieved a response rate of 14.73% (1971/13,384) (state ranges 10.1%–20.3%). This response rate is in keeping with other parent-oriented email-based surveys, and, coupled with its lack of incentive for participation, is within an acceptable range for this population [19]. As Figure 1 illustrates, of the 13,384 individuals solicited, 740 had email addresses on record that were no longer in use or invalid. A small number (n = 142) chose to opt out of the survey using an embedded link within the email solicitation to remove their name and email address from the mailing list. There were 20 respondents who were contacted inappropriately (in a majority of cases these were a school counselor listed as a child’s contact) and 2 who did not want to complete the survey online. Five more recipients had technical difficulties precluding their ability to fill out the survey. We excluded an additional 23 respondents, as they filled out the survey but stated that they were not the parent of the child or that their child had not yet taken an online course.

### Survey Design and Measures

All members of the study team contributed to the design and pilot testing of this survey for parents that would take about 5 minutes to complete. The survey was constructed with the following domains: basic demographics and educational history of the child, parental education, the CSHCN Screener [20], and the child’s experiences and educational achievement with online class(es). We purposefully chose the CSHCN Screener to bridge medical and educational outcomes. Traditionally, educationally based screeners cite disabilities that reflect those conditions that will directly require adaptive learning tools, such as learning disabilities, emotional disturbances, or speech and language impairments [17]. The CSHCN Screener uses a comprehensive approach to health and is a well-validated and reliable tool aimed at discriminating those children who have an ongoing health need [21,22]. Although brief, the self-administered CSHCN Screener consists of five consequences-based questions that identify children with chronic or special health care needs [22]. These health consequences are summed into three nonexclusive definitional domains: dependency on prescription medications, service use above that considered usual or routine, and functional limitations [22]. As a reference, roughly 15% of the general public and 20.7% of those enrolled in Medicaid screen positive using this screener [23]. We conducted all univariate, bivariate, and multivariate analyses with Stata version 9.2 (StataCorp LP, College Station, TX, USA). The accepted level of significance was *P *< .05.

## Results

### Demographics of Participants in Online Schooling

This survey reports on 1971 parents of any students of online schools in three US states. Table 1 shows demographic comparisons of survey participants and available data from face-to-face public-school classrooms in each state. The students were overwhelmingly older adolescents (86.90%), ages 15–18 years. There was also a high prevalence of CSHCN (476/1971, 24.6% overall, range 21.0%–29.9%), which is significantly greater than in the general population (15.0%–15.4%; see also Table 2) [23]. Overall and within each state, online-school parents participating in this survey were more likely than parents of students statewide in traditional schools to report having a bachelor’s degree or higher, and more of them reported that their child was female (1138/1971, 57.88%) or white (1397/1971, 70.88%). While there was variation by state, overall there were fewer black and Hispanic students and more children of other or mixed races and ethnicities than in traditional schools. Parents of online-school students rated their child’s educational success as “very good” or “excellent” 62.9% (846/1971) of the time, yet there was a wide range by state (52.7%–70.0%). Importantly, there was no difference in the distribution of reported grades (recorded as A, B, C, D, or failing) between their child’s online-school grade and their usual grades from traditional school.

**Table 1:** Demographics of students in online school and traditional school according to parental report.

Characteristic		Total	State 1		State 2		State 3	
		Online school population (n = 1971)	Online school population (n = 553)	Traditional school population	Online school population (n = 831)	Traditional school population	Online school population (n = 593)	Traditional school population
**Gender**								
	Female, n (%)	1138 (57.88%)	312 (56.4%)	NA^a^	503 (61.2%)	NA	323 (54.6%)	NA
**Age distribution (years), n (%)**								
	≤14	195 (9.9%)	40 (7%)	NA	114 (13.8%)	NA	41 (7%)	NA
	15–18	1711 (86.90%)	473 (85.7%)	NA	704 (85.4%)	NA	534 (90.1%)	NA
	19+	63 (3%)	39 (7%)	NA	6 (1%)	NA	18 (3%)	NA
**Race/ethnicity, n (%)**								
	White	1397 (70.88%)	327 (59.1%)	46.5%^b^	607 (73.4%)	55.7%^b^	463 (78.1%)	53.9%^b^
	Hispanic	50 (3%)	5 (1%)	1.7%^b^	34 (4%)	9.3%^b^	11 (2%)	4.6%^b^
	Black	367 (18.6%)	188 (34.0%)	50.8%^b^	89 (11%)	28.3%^b^	90 (15%)	39.8%^b^
	Other/mixed	157 (8.0%)	33 (6%)	1.0%^b^	95 (12%)	3.6%^b^	29 (5%)	1.7%^b^
**Health, n (%)**								
	Child with a special health care need^c^	476 (24.6%)	116 (21.0%)	15.0%^d^	193 (23.2%)	15.4%^d^	173 (29.9%)	15.2%^d^
**Parental education, n (%)**								
	Bachelor’s degree or higher	967 (49.3%)	254 (46.0%)	25.6%^e^	440 (53.8%)	25.6%^e^	273 (53.8%)	22.6%^e^
**Educational success in past 6 months, n (%)**								
	Very good/excellent	846 (62.9%)	607 (59.5%)	NA	555 (70.0%)	NA	291 (52.7%)	NA
	Poor/fair/good	499 (37.1%)	215 (40.5%)	NA	238 (30.0%)	NA	261 (47.3%)	NA

^a ^Not available.

^b ^Data from Schooldatadirect.org, an online service of the State Education Data Center, an initiative of the Counsel of Chief State School Officers, funded by the Bill and Melinda Gates Foundation.

^c ^Children with a special health care need were defined using the CSHCN Screener.2.

^d ^Child and Adolescent Health Measurement Initiative. 2005/2006 National Survey of Children with Special Health Care Needs, Data Resource Center for Child and Adolescent Health website. Retrieved November 3, 2009 from www.cshcndata.org.

^e ^Adults aged 25 years or older. Data from http://www.census.gov/population/www/socdemo/education/cps2006.html.

**Table 2:** Domains of the Children with Special Health Care Needs (CSHCN) Screener.

Domain		Total online school population (n = 1971)
Overall: having a special health care need^a^		476 (24.6%)
Definitional domains		
	Dependency on prescription medications	400 (21.7%)
	Service use above that considered usual or routine	211 (10.8%)
	Functional limitations	119 (6.2%)

^a ^The domains are not mutually exclusive categories, as a child identified by the CSHCN Screener can qualify on one or more definitional domains [16].

 Parents reported diverse reasons why their children took online classes. The majority (377/497, 76.0%) took online classes because their school did not offer the class, they wanted to augment their education, or they had scheduling difficulties. Only 16% (78/497) took classes because of disciplinary concerns or credit recovery. Only a small portion (41/497, 8%) cited health or significant social concerns as the reason for taking online classes.

### Educational Success in Online Compared With Traditional Schools

Matching a child’s reported online-school grade with their usual grade in a traditional class was possible in 61.09% of all study participants (1204 parents report their child’s grades, since not all had completed the course or could predict their child’s grade). When comparing reported grades from traditional or online schools, certain populations consistently reported low performance. Boys, black children, and CSHCN had significantly lower grades (as demonstrated by nonoverlapping confidence intervals of grades compared with girls; white, Hispanic, and others; and healthy children, respectively; Table 3). Comparisons between traditional and online schools yielded no differences, even within subgroups. Online schools and traditional schools seem to have similar success rates within populations in straight, unadjusted comparisons.

**Table 3:** Parental report of grades^a ^in traditional and online school by demographic characteristics.

Characteristic		Traditional school grade (n = 1386)		Online school grade (n = 1207)	
		Mean	95% CI^b^	Mean	95% CI
Female		3.37	3.32–3.41	3.34	3.28–3.41
Male^c^		2.93	2.87–3.01	3.08	2.99–3.17
**Race/ethnicity**					
	White	3.24	3.19–3.29	3.30	3.23–3.36
	Black^c^	2.84	2.74–2.94	2.82	2.70–2.96
	Hispanic	3.33	3.09–3.57	3.31	2.99–3.63
	Other/mixed	3.43	3.29–3.57	3.42	3.26–3.58
Children with special health care needs^c^		2.93	2.83–3.02	3.02	2.91–3.14
No special health care needs		3.27	3.22–3.31	3.30	3.24–3.35

^a ^Grade point average equivalences: A = 4.0; B = 3.0; C = 2.0; D = 1.0.

^b ^Confidence interval.

^c ^Significantly different reported grades (*P *< .0001) between males and females, blacks and all others, and children with special health care needs and those without within traditional or online-school classes. No significant differences in this relationship are seen between traditional and online grades.

 However, multivariate regression techniques that adjust for known educational performance factors further clarified these univariate trends to help decipher the question of whether children in high-risk groups performed better in online or in traditional classes. Controlling for age, gender, race, and parental education, CSHCN and black children were significantly *more *likely to have lower grades in online classes than their usual grades in traditional classrooms (adjusted odds ratio [aOR] 1.45, 95% confidence interval [CI] 1.29–1.62 for CSHCN, *P *< .001; aOR 2.73, 95% CI 2.11–3.53 for black children, *P *< .001). In contrast, children whose parents had a bachelor’s degree or higher were more likely to perform better in online schools (aOR 1.45, 95% CI 1.15–1.82, *P *< .001).

## Discussion

Child health care providers frequently encounter children who fall behind in their education because of health, behavioral, or situational concerns that may inhibit their ability to learn in a traditional classroom setting [7,24]. Outside of individualized education programs or 504 plan adaptations [6], United States physicians are generally without tools to support families with struggling children. Online schooling offers a unique means with which to maintain educational progress in order to satisfy the myriad needs that children may have. This study, even using parent-reported grades, supports previous work demonstrating that online schools offer an equivalent educational experience. As such, providers might choose to recommend online schools, since they can provide an educational choice for medically or socially challenged children instead of traditional schools with strict rules such as attendance requirements [12,25,26]. This study confirms that significantly more students who have special health care needs are opting into this online educational opportunity. Importantly, however, while there are diverse students attending online schools, their demographics do not mirror those of the general population, an observation that requires attention from the administrators of online schools. Further, despite the potential of online schools to address specific needs, this study confirms through adjusted analyses that children at high risk for poor health and educational outcomes do not improve their poor educational performance in online schools.

 Few studies have linked health and educational outcomes [27], likely due to the few means of measurement, as well as the different functional definitions of “at risk” that each discipline uses. Students who meet definitions for needing special education may or may not screen positive in the CSHCN Screener (as demonstrated in the distribution of positive findings in the screener; Table 2). This study highlights this research gap for health and education, and underscores the need to develop linked, efficiently defined, and codependent health and education outcomes, given that many social forces affect both health and education, such as poverty and family structure [24,28]. To achieve educational success, children must have sufficient health to learn, and, in the reverse, children with educational failures may well experience poorer health.

This study leads to the hypothesis that improvements in health outcomes may result from educational success, especially through novel educational opportunities and modalities like online schools. Few studies have examined the intertwined relationship between health and educational achievement, although the parallel sources of literature reveal the same populations having poor outcomes [24,27-30]. The most informative studies have prospectively followed medically complex birth cohorts (such as cancer survivors or prematurely born infants) and have monitored intellectual progress [3,31]. There are otherwise few opportunities to link medically oriented pediatric datasets, such as vital statistics, to those collected by the US Department of Education. Other studies use more readily available intermediate outcomes, such as the number of school days missed or school-related quality-of-life indicators, as reported through the PedsQL, to measure the impact of health on daily functioning [32]. These indicators, although important, lack any assessment of educational success itself. Future studies may seek to identify the crossover between the term *disability *as commonly used in education and a designation used in clinical settings such as “having a special health care need.” Based on this large population of CSHCN who seek online schools and the equivalent education that online schooling can offer, online schools may carry the dual role of enhancing educational progress and improving health outcomes if designed to maximize opportunities for child with special needs.

This study confirms that, while online schools may provide equivalent education, it does not help at-risk populations. More comprehensive screening measures for children who participate in online-schooling courses may be necessary to confirm the presence of individuals with special health care needs. Indeed, success factors associated with online schooling built by Cavanaugh [33] and others and adapted by Black et al [34] suggest that a student’s abilities and disabilities predict online-schooling success. However, in these studies and others, students’ abilities and disabilities were conceptualized from a cognitive perspective rather than from a physical health perspective [17]. Predictive screening has been proposed by Roblyer et al [35] and supported by Black et al [34] as a means of identifying individuals who may need more academic attention. As yet, a valid and reliable tool for assessment has not been developed.

 Not only do online schools maintain educational achievement gaps in certain populations known to have both poor health and educational outcomes [4,36], they may also cause further disparities in these populations. The multivariate regressions imply that these students perform significantly worse academically in online schools than white, healthy students. That these disparities persist in an online world where teachers may not know any physical characteristics of their students suggests that these disparities are complex and will not be remedied by simplistic solutions. Many online courses, similar to other forms of online content [37], are typically not built with accessibility standards in mind. In fact, few US K-12 online schools have protocols in place to accurately identify students with differing abilities [38]. Future research can, however, use these two divergent settings (online and traditional classrooms) to perform comparative-effectiveness research techniques to seek methods that may reduce health and educational disparities [39]. In addition, directly recruiting children from special populations (such as those with special heath care needs) into online schooling will provide the opportunity to measure whether, with direct assistance, they can gain higher educational progress. It is possible that designing courses that are compliant with the Americans with Disabilities Act guidelines will offer benefit to students with special health care needs. To this end, Repetto et al [16] offer several pedagogical strategies to assist individuals with specific needs in online courses, including being flexible with assignments and learning modalities; connecting content with real-world examples that would be salient to the student’s specific context; mentoring; and offering professional development for online instructors. Health and education are linked, codependent outcomes. Studies of international comparisons of online-school systems or measures of online-school integration into traditional schools may provide additional methods to calculate the achievement of high-risk populations.

 This study has several limitations that merit comment. First, our surveys experienced low response rates in each state. That said, Internet-based research is emerging as a powerful means to communicate with parents, and we believe this response rate is sufficient to provide a meaningful summary of their points of view, and we feel that any incentive for response may have its own inherent bias [19]. Second, the generalizability of the sample is unclear given the high proportion of highly educated parents. We do not know whether a non-Internet-based survey would attract a more representative sample of parents, although other forums such as parent–teacher meetings have a notoriously skewed sample of parents. Finally, the achievement data used in this study presented a limitation. We used parent-reported grade data, which is subject to recall and social desirability bias. In addition, we used course grades as a measure of student achievement. Course grades are a subjective, nonstandardized means of assessing student performance. Further, we did not stratify grade data based on course subject matter. Finally, the population of students who use online schools may actually be biased toward the higher-educated families, requiring these schools to better market their opportunities in all school settings.

 Health care providers need to be aware of the technological and pedagogical advances during the past 10 years that have enabled primary and secondary students through state-accredited online schools the opportunities for “any time, any place, any pace” learning. Online schooling is an equivalent and novel means with which to maintain educational progress. However, it also provides an unmet opportunity to narrow the achievement gap for CSHCN and children in high-risk populations such as racial and ethnic minorities. As online-schooling programs become more prominent, accepted, and popular, it is imperative to adapt online instruction to populations known to experience less educational success, such as racial and ethnic minorities and those with special health care needs. Given the affordances of online schools and the potential to follow children in the long term with health conditions through school, the health, well-being, and long-term successes for children who are at high risk for poor educational and health outcomes may yet be improved.

## Abbreviations

aOR: adjusted odds ratio
CI: confidence interval
CSHCN: children with special health care needs
K-12: kindergarten to 12th grade

## References

[1] Yin HS, Johnson M, Mendelsohn AL, Abrams MA, Sanders LM, Dreyer BP (2009). The health literacy of parents in the United States: a nationally representative study. Pediatrics.

[2] DeWalt DA, Hink A (2009). Health literacy and child health outcomes: a systematic review of the literature. Pediatrics.

[3] Lancashire ER, Frobisher C, Reulen RC, Winter DL, Glaser A, Hawkins MM (2010). Educational attainment among adult survivors of childhood cancer in Great Britain: a population-based cohort study. J Natl Cancer Inst.

[4] Taras H, Potts-Datema W (2005). Chronic health conditions and student performance at school. J Sch Health.

[5] National Asthma Education and Prevention Program--School Subcommittee; National School Boards Association; American School Health Association; American Diabetes Association; American Academy of Pediatrics; Food Allergy and Anaphylaxis Network; Epilepsy Foundation (2003). Students with chronic illnesses: guidance for families, schools, and students. J Sch Health.

[6] American Academy of Pediatrics, Committee on Children with Disabilities (1999). The pediatrician's role in development and implementation of an Individual Education Plan (IEP) and/or an Individual Family Service Plan (IFSP). Pediatrics.

[7] Kelly DP, Aylward GP (2005). Identifying school performance problems in the pediatric office. Pediatr Ann.

[8] Bravender T (2008). School performance: the pediatrician's role. Clin Pediatr (Phila).

[9] Barbour M, Brown R, Waters LH, Hoey R, Hunt JL, Kennedy K, Ounsworth C, Powell A, Trimm T (2011). Online and Blended Learning: A Survey of Policy and Practice of K-12 Schools Around the World.

[10] Greenway R, Vanourek G (2006). The virtual revolution: understanding online schools. Educ Next.

[11] Watson J, Gemin B, Ryan J (2008). Evergreen Consulting Associates.

[12] Cavanaugh C, Gillan KJ, Kromrey J, Hess M, Blomeyer R (2004). Learning Point Associates.

[13] Dickson WP (2005). Toward a deeper understanding of student performance in virtual high school courses: using quantitative analyses and data visualization to inform decision making. Smith R, Clark T, Blomeyer RL, editors. A Synthesis of New Research in K-12 Online Learning.

[14] Russell TL (1999). The No Significant Difference Phenomenon.

[15] Bernard RM, Abrami PC, Lou Y, Borokhovski E, Wade A, Wozney L, Wallet PA, Fiset M, Huang B (2004). How does distance education compare with classroom instruction? A meta-analysis of the empirical literature. Rev Educ Res.

[16] Repetto J, Cavanaugh C, Wayer N, Liu F (2010). Virtual high schools: improving outcomes for students with disabilities. Q Rev Distance Learn.

[17] Müller E (2010). Project Forum.

[18] (2009). AT&T.

[19] Manfreda KL, Bosnjak M, Berzelak J, Haas I, Vehovar V (2008). Web surveys versus other survey modes: a meta-analysis comparing response rates. Int J Market Res.

[20] Bethell CD, Read D, Stein RE, Blumberg SJ, Wells N, Newacheck PW (2002). Identifying children with special health care needs: development and evaluation of a short screening instrument. Ambul Pediatr.

[21] Child and Adolescent Health Measurement Initiative.

[22] Bethell CD, Read D, Blumberg SJ, Newacheck PW (2008). What is the prevalence of children with special health care needs? Toward an understanding of variations in findings and methods across three national surveys. Matern Child Health J.

[23] Carle AC, Blumberg SJ, Poblenz C (2011). Internal psychometric properties of the Children with Special Health Care Needs Screener. Acad Pediatr.

[24] Sanders LM, Federico S, Klass P, Abrams MA, Dreyer B (2009). Literacy and child health: a systematic review. Arch Pediatr Adolesc Med.

[25] Cavanaugh C (2009). Effectiveness of cyber charter schools: a review of research on learnings. TechTrends.

[26] Ferdig RE, DiPietro M, Papanastasiou E (2005). Teaching and Learning in Collaborative Virtual High Schools.

[27] Forrest CB, Bevans KB, Riley AW, Crespo R, Louis TA (2011). School outcomes of children with special health care needs. Pediatrics.

[28] Byrd RS, Weitzman ML (1994). Predictors of early grade retention among children in the United States. Pediatrics.

[29] Fiscella K, Kitzman H (2009). Disparities in academic achievement and health: the intersection of child education and health policy. Pediatrics.

[30] Haas SA, Fosse NE (2008). Health and the educational attainment of adolescents: evidence from the NLSY97. J Health Soc Behav.

[31] Chyi LJ, Lee HC, Hintz SR, Gould JB, Sutcliffe TL (2008). School outcomes of late preterm infants: special needs and challenges for infants born at 32 to 36 weeks gestation. J Pediatr.

[32] Wade TJ, Mansour ME, Line K, Huentelman T, Keller KN (2008). Improvements in health-related quality of life among school-based health center users in elementary and middle school. Ambul Pediatr.

[33] Cavanaugh C (2004). Distance education success factors. Khosrow-Pour M, editor. Encyclopedia of Information Science and Technology.

[34] Black EW, Ferdig RE, DiPietro M (2008). An overview of evaluative instrumentation for virtual high schools. Am J Distance Educ.

[35] Roblyer MD, Davis L, Mills SC, Marshall J, Pape L (2008). Toward practical procedures for predicting and promoting success in virtual school students. Am J Distance Educ.

[36] Rose RM, Blomeyer RL (2007). North American Council for Online Learning.

[37] Zeng X, Parmanto B (2004). Web content accessibility of consumer health information web sites for people with disabilities: a cross sectional evaluation. J Med Internet Res.

[38] Black EW, Thompson LA, Ashkenazy N, Ferdig RE, Kisker T (2010). As assessment of the health of virtual school participants and an innovative outreach program for chronically ill children.

[39] Committee on Comparative Effectiveness Research Prioritization, Institute of Medicine (2009). Initial National Priorities for Comparative Effectiveness Research.

